# Defective Ribosome Recycling: A Bridge Between Translation Fidelity, Organelle Dysfunction, and Diseases: Exploring How Flawed Ribosome Recycling Factors Impact Ribosome Metabolism and Organelle Homeostasis, Leading to Human Diseases

**DOI:** 10.1002/bies.70054

**Published:** 2025-08-11

**Authors:** Foozhan Tahmasebinia, Zhihao Wu

**Affiliations:** ^1^ Department of Biological Sciences Dedman College of Humanities and Sciences Southern Methodist University Dallas Texas USA

**Keywords:** 40S subunit recycling, cancer, CAT‐tailing, mitochondria, neurodegenerative diseases, ribosome‐associated quality control (RQC), ribosome recycling

## Abstract

Ribosome recycling is a fundamental biological process crucial for cellular health. Defective recycling disrupts ribosome biogenesis and organelle function, particularly in mitochondria, contributing to ribosomopathies, neurodegenerative diseases, and cancer. While not directly linked to human diseases via known genetic mutations, emerging evidence suggests a critical interplay between ribosome recycling and organelle quality control. Impaired ribosome recycling leads to aberrant ribosome production, compromised translational quality control, protein misfolding, and subsequent organelle dysfunction and cellular stress. These cascading defects underscore the critical need for effective ribosome reutilization, especially under stress, as disruptions can cause translational arrest and heightened stress signaling, perturbing cellular homeostasis. Our analyses establish an indirect but significant link between ribosome recycling and human disease, offering new perspectives on how translational fidelity and organelle maintenance converge to support cellular well‐being.

## Graphical abstract

The Role of Ribosome Recycling in Human Diseases

This diagram highlights how disruptions in ribosome translation and recycling can negatively impact cellular and organismal health. Usually, these processes operate efficiently to maintain cellular protein homeostasis. However, issues like translation stalling or ribosome collisions can lead to various pathological conditions.

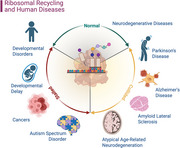

## 1 Introduction: Ribosome Recycling, a Cellular Event Beyond Our Imagination

Ribosomes, ubiquitous organelles in eukaryotic cells, are fundamental to protein translation, a process underpinning all cellular activities. The study of ribosome metabolism has a long history, but until recently, new discoveries have continued to expand our knowledge in this area [1]. During a complete protein translation cycle, ribosomes bind to mRNA and go through the following three stages: initiation, elongation, and termination [2, 3]. Ribosome recycling is a key step in this cycle, ensuring the disengagement of ribosomal large and small subunits for subsequent rounds of protein synthesis [1, 4]. This process becomes even more vital when translation is unexpectedly halted [5], as it prevents stalled ribosomes from blocking the upstream peers, allowing translation to continue [6, 7]. The iterative nature of ribosome recycling is essential for cellular efficiency and viability, enabling hundreds of recycling events per cell cycle to support the synthesis of vital proteins, including those necessary for their own replacement [8, 9]. Consequently, disruptions in recycling efficiency can significantly impact polypeptide synthesis, altering cellular gene expression and proteostasis. Recent studies indicate that ribosome recycling intersects with various signaling pathways, particularly those related to energy metabolism and organelle quality control [10, 11, 12].

Despite its importance, no monogenic human disorders are currently attributed to mutations in essential recycling factors like *ABCE1* or *PELO*. This suggests that, while indispensable, their disruption may only cause disease under specific cellular conditions or with additional stressors. In contrast, mutations in ribosome‐associated quality control (RQC) components, such as NEMF, are mechanistically tied to neurodevelopmental disorders [13]. While correlative research implicates the involvement of *ABCE1* and *PELO* in neurodegenerative diseases, direct causal evidence remains elusive. Certain tissues, notably the nervous system, may be more susceptible to ribosome recycling disruptions due to limited local ribosome availability. For instance, translation near neuronal synapses is more restricted than in somatic regions [14], and impaired ribosome recycling has been associated with numerous neurological disorders [15, 16]. Cancer cells also exhibit heightened sensitivity to ribosome recycling efficiency, given their elevated demand for protein synthesis [17]. Under cellular stress, such as viral infection or nutrient deprivation, efficient ribosome recycling is critical for sustaining protein synthesis and organelle function [2, 18]. Investigations into the links between ribosome recycling and human disease reveal a complex landscape characterized by indirect associations and persistent research gaps. The following discussion will delineate the sequential process of ribosome cycling, elucidate the fate of each component within the ribosome cycling complex, and summarize the potential consequences of ribosome cycling errors.

## 2 Rondeau of Translation: Ribosome Recycling in Normal Termination

### 2.1 Two Worlds of Translation: A Comparison of Mitochondrial and Cytoplasmic Ribosomes

Eukaryotic cells possess the following two independent protein translation systems: cytoplasmic and mitochondrial, with an additional distinct mechanism in plant chloroplasts [19]. Mitochondrial ribosomes, resembling bacterial ribosomes, are believed to have originated from an endosymbiotic relationship between proto‐eukaryotic cells and α‐proto‐bacteria [5, 20]. This evolutionary divergence has led to significant differences in size, structure, and molecular composition between the two ribosomal types. While both execute peptide bond formation, their architectures are adapted to distinct cellular environments [21]. Ribosome size, measured in Svedberg units (S), is a key differentiator. Eukaryotic cytoplasmic ribosomes are conserved 80S particles (80S: 60S large, 40S small subunits) [21, 22]. In contrast, mitochondrial ribosomes display remarkable diversity across lineages. Mammalian mitochondrial ribosomes are notably smaller (55S: 39S large, 28S small subunits) [21, 23]. However, yeast (*Saccharomyces cerevisiae*) mitochondrial ribosomes are larger (74S: 54S large, 37S small subunits), and flowering plant mitochondrial ribosomes are larger still (78S–80S), rivaling their cytoplasmic counterparts [24, 25]. This variability underscores diverse evolutionary strategies, with mammals favoring size minimization and fungi/plants exhibiting expansion.

Compositionally, bacterial and eukaryotic cytoplasmic ribosomes are RNA‐based machines with an approximate 1:2 protein‐to‐RNA mass ratio [21, 26]. Mammalian mitochondrial ribosomes, however, are protein‐rich with a 2:1 ratio [26]. This shift is attributed to “reductive” evolution (rRNA shortening/loss) and “constructive” evolution (acquisition of mitochondria‐specific proteins) [24]. These differences are summarized in Table 1. The ribosomal systems also differ profoundly in their mechanisms of action, especially translation initiation, having evolved distinct strategies for mRNA recognition and start codon selection [26]. While a detailed exploration of this complex topic is beyond the scope of this article, recent studies have highlighted the divergent recycling mechanisms resulting from these structural differences.

**TABLE 1:** Comparative structural and compositional features of ribosomes.

Feature	E. coli (bacteria)	Eukaryotic cytoplasm	Mammalian mitochondria	Yeast mitochondria	Plant mitochondria
Overall sedimentation	70S	80S	55S	74S	∼78S–80S
LSU/SSU sedimentation	50S/30S	60S/40S	39S/28S	54S/37S	∼50S/∼33S
Small subunit rRNA	16S	18S	12S	15S	18S
Large subunit rRNAs	23S, 5S	28S, 5.8S, 5S	16S, tRNA^Val^	21S	26S, 5S
Total protein count	∼55	∼80	∼82	∼84	>80
Protein:RNA ratio	∼1:2	∼1:1.5	∼2:1	∼1:1	Protein‐rich

### 2.2 Ribosome Recycling in the Cytoplasm

The eukaryotic ribosome cycle encompasses the following two steps: (1) the detachment of the 60S large subunit from the 40S small subunit, and (2) the separation of the 40S subunit from the mRNA and deacetylated tRNA. This process initiates with the ribosome recognition of a stop codon at the A site, mediated by a heterodimeric complex of eukaryotic release factor 1 (eRF1) and eukaryotic release factor 3 (eRF3). eRF1 serves as the pivotal protein in eukaryotic translation termination, exhibiting the capacity to recognize all three stop codons (UAA, UAG, UGA) within the ribosomal A‐site. Furthermore, eRF1 catalyzes the hydrolysis of the ester bond connecting the nascent polypeptide to the P‐site tRNA [27, 28]. Structurally, eRF1's three‐dimensional conformation exhibits striking similarity to that of a tRNA molecule, enabling its precise accommodation within the A‐site, a ribosomal locus typically occupied by aminoacyl‐tRNAs during translational elongation [28]. eRF3 functions as an indispensable, ribosome‐dependent GTPase that collaborates with eRF1 during the termination of translation. Its primary function involves harnessing the energy derived from GTP hydrolysis to enhance both the rate and fidelity of the termination process [28]. During translation termination, the eRF1‐eRF3 complex facilitates the formation of a post‐termination 80S ribosome complex, comprising the 80S ribosome, mRNA, and deacetylated tRNA at the P site. This intricate mechanism ensures the accurate cessation of translation and primes the ribosome for subsequent recycling.

#### 2.2.1 Dissociation of the 80S Complex

The initial step in ribosome recycling involves the dissociation of the 80S ribosomal complex into its 40S and 60S subunits. This critical separation is primarily mediated by the ATP‐binding cassette protein Rli1/ABCE1 (yeast/human nomenclature), as supported by both in vitro and in vivo evidence [29, 30, 31, 32]. Rli1/ABCE1 is characterized by an iron‐sulfur (Fe‐S) cluster domain that interacts with eRF1 [33, 34], and two nucleotide‐binding domains (NBDs) that bind ATP and connect to the ribosome's GTPase center [35, 36]. While Rli1/ABCE1 utilizes ATP hydrolysis to facilitate subunit separation via eRF1, the precise mechanism remains unclear [31, 32]. Intriguingly, the “power stroke” for splitting is not driven by ATP hydrolysis but by ATP binding. ATP binding induces a significant conformational change in ABCE1, transitioning it from an open to a closed, ATP‐occluded state [36]. This triggers a dramatic 150° rotation of the Fe‐S cluster domain, which swings out from a cleft between the NBDs and acts as a physical wedge, prying the 40S and 60S subunits apart by disrupting critical inter‐subunit bridges [37]. Other eukaryotic initiation factors, such as eIF3, eIF1, eIF1A, and eIF3j, have limited effectiveness in this process, although eIF3j slightly influences Rli1/ABCE1's ATPase activity [38, 39].

#### 2.2.2 Recycling the 40S Subunit

Following dissociation, the 40S subunit must release the mRNA and deacetylated tRNA located in the P‐site. This final recycling step, which disassembles the post‐termination 40S complex, is accomplished by a network of factors known as the Tma (Translation machinery‐associated) proteins: the heterodimer Tma20:Tma22 (mammalian orthologs MCT‐1:DENR) and the monomeric protein Tma64 (mammalian ortholog eIF2D) [40, 41]. Their collective function is to promote the dissociation of the post‐termination 40S complex, thereby releasing the tRNA and mRNA and freeing the 40S subunit for a new round of initiation. Experiments in yeast and HeLa cells have shown that without these proteins, 80S ribosomes accumulate at one ribosome length upstream of the stop codon, while 40S subunits stack at the stop codon, unequivocally demonstrating their involvement in recycling [42, 43, 44].

In yeast, the Tma20:Tma22 complex is the primary regulator, with Tma64 supporting it [44]. Structurally, Tma64/eIF2D has five domains, including an eIF1 (SUI1) domain; likewise, Tma20 and Tma22 resemble various parts of Tma64, with Tma22 also containing an eIF1 domain [41]. Interestingly, while Rli1/ABCE1 is considered essential in both yeast and human cells, the 40S recycling factors are not deemed critical in yeast, indicating the existence of alternative 40S subunit recycling pathways, such as through eIF1 or a spontaneous dissociation mechanism [45]. In mammals, the regulatory logic appears more intricate. The MCTS1:DENR complex has been shown to actively promote reinitiation following the translation of specific short upstream open reading frames (uORFs) [46]. The mammalian eIF2D, in contrast, does not seem to function as a general reinitiation factor, and its depletion leads to distinct, widespread changes in gene expression, indicating a significant divergence in the functions of these two systems [46]. This functional divergence highlights how a fundamental piece of cellular machinery can adapt to different regulatory strategies across evolution.

This recycling factor network should not be regarded merely as a housekeeping “clean‐up crew.” Instead, it represents a critical regulatory hub that governs the choice between complete termination and reinitiation. By controlling the fate of the post‐termination 40S subunit, these factors directly influence the expression of thousands of genes regulated by uORFs. This positions them as key modulators of proteome plasticity, enabling cells to execute complex gene expression programs in response to developmental cues and environmental stress.

### 2.3 Ribosome Recycling in Mitochondria

Mitochondrial ribosome recycling is more reminiscent of that found in eubacteria. During translation termination, stop codons within the open reading frame (ORF) are recognized by release factors RF1 or RF2 [47, 48, 49, 50, 51]. This recognition triggers a conformational change in the release factor, leading to hydrolysis and release of the nascent protein [47, 48, 49, 51]. Release factor 3 then assists in the displacement of RF 1 and RF 2 from the ribosome [52, 53]. Subsequently, elongation factor G (EF‐G) and ribosome recycling factor (RRF) then collaborate to disassemble the terminated 70S ribosome into individual subunits [54, 55, 56]. RRF acts as a structural mimetic of tRNA [57, 58]. Despite cryo‐EM structural data, the precise role of EF‐G in this recycling process remains uncertain due to resolution constraints [59, 60]. HflX, a universally conserved GTPase, primarily functions in bacteria as a ribosome‐splitting factor. Its upregulation and activation occur under various stress conditions, including heat shock [61], antibiotic exposure [62, 63, 64], and manganese imbalance [65, 66]. HflX binds to the ribosomal E‐site, and its GTPase activity is allosterically modulated by the state of the Peptidyl Transferase Center (PTC) and the nascent peptide exit tunnel, rather than the A‐site [67]. The splitting action involves the N‐terminal domain of HflX penetrating the PTC, inducing significant ribosomal conformational changes, disrupting intersubunit bridges, and ultimately prying the subunits apart [67].

Eukaryotic mitochondria exhibit functional specialization, particularly concerning ribosome recycling. GTPBP6, similar to HflXd, acts as a ribosome recycling factor utilized during mitochondrial stress [68, 69]. Additionally, mitochondria‐specific RRF and EF‐G coordinate to recycle mitochondrial ribosomes [50, 70, 71]. Mammalian mitochondria have two EF‐G homologs, EF‐G1mt and EF‐G2mt, both demonstrating substantial ribosome‐dependent GTPase activity [72]. Unlike bacterial EF‐G, which has dual roles in elongation and recycling, eukaryotic mitochondrial EF‐G1mt (encoded by *GFM1*) is solely a translocase, facilitating GTP‐dependent tRNA movement during elongation. In contrast, EF‐G2mt (encoded by *GFM2*, also known as RRF2mt) has specialized as a ribosome recycling factor, losing its translocation activity. It collaborates with mitochondrial Ribosome Recycling Factor (RRFmt) to disassemble the post‐translational 55S mitochondrial ribosome [72]. This functional divergence between EF‐G1mt and EF‐G2mt represents a powerful evolutionary strategy. By separating elongation and recycling into two distinct proteins, the cell achieves more sophisticated and independent regulation of these crucial phases of mitochondrial protein synthesis, minimizing conflicts and optimizing each factor for its specific task. This specialization, achieved through gene duplication, is a recurring theme in the evolution of complex cellular machinery.

## 3 Imperfect Cadence: Ribosome Recycling in Abnormal Termination

The translation process can be easily decelerated or even stalled by mRNA intrinsic features (e.g., suboptimal codons, long poly(A) sequences/no stop codon [73, 74], higher‐order structures [75]) and extrinsic factors (e.g., mRNA truncations [76], inadequate aminoacyl‐tRNA availability, ultraviolet irradiation) [77, 78]. Eukaryotic cells employ the ribosome‐associated quality control (RQC) mechanism to detect ribosomal collisions stemming from stalled translation [13]. The RQC pathway is crucial for surveying and clearing stalled ribosomes, thereby preventing the build‐up of aberrant proteins [79]. A critical aspect of RQC is the disassembly and recycling of ribosomes, with different details broadly categorized into mitochondrial and cytosolic RQCs.

Research into mitochondrial ribosome‐associated quality control (mtRQC) is relatively scarce, and the existence of an evolutionarily conserved RQC mechanism within mitochondria has long been debated [80]. This uncertainty stems from the fact that mitochondrial ribosomes exclusively translate genes encoded by mitochondrial DNA (mtDNA), which comprises only 37 genes encoding 13 proteins. Consequently, mitochondria, even when compared to their ancestral counterparts, appear to lack the inherent evolutionary pressure to maintain an extensive suite of translation quality control systems. Nevertheless, despite the absence of a mechanism akin to that found in eubacteria [81], cryo‐electron microscopic observations of stalled mitochondrial ribosomes have provided valuable insights [82]. The stalled mitochondrial 39S large ribosomal subunit complex includes a peptidyl‐tRNA that associates with a heterodimer of the release factor homolog C12orf65 (mitochondrial Release Factor in Rescue or “mtRF‐R”) and MTRES1, a double‐stranded RNA binding protein. The mtRF‐R/MTRES1 complex aids in the dissociation of nascent polypeptide chains and peptidyl transfer RNA from arrested mitochondrial ribosomes. Although mechanistically different, the mtRF‐R/MTRES1 complex exhibits effects analogous to the Vms1/ANKZF1 pathway of cytosolic RQC [82]. Mitochondria rely on a specialized ribosome quality control system to maintain translation fidelity, likely due to the frequent occurrence of mitochondrial ribosome stalling resulting from faulty mt‐tRNA polyadenylation. Therefore, most studies on mitochondrial translation quality control have focused on the import regulation of cytosolic‐synthesized mitochondrial proteins (encoded by the nuclear genome) [80]. RQC on the mitochondrial outer membrane (also known as MISTERMINATE) is essentially a form of cytosolic RQC and is discussed in conjunction with it [78].

The RQC pathway involves a series of well‐defined steps as follows:

- Recognition of stalled or collided ribosomes:

Ribosome stalling is commonly due to translation issues like mRNA truncation, lack of charged tRNAs, or poly(A) tail translation. Stalling does not always involve ribosome collisions (e.g., 3'‐end stalling). This process requires the mRNA surveillance function of Pelota/PELO, which recruits the PELO:HBS1L rescue factor complex. This complex then recruits ATP‐binding cassette subfamily E member 1 (ABCE1) to split the 80S ribosome into subunits for further processing [9]. Pelota (PELO in mammals, Dom34 in yeast) is structurally similar to the canonical termination factor eRF1 but functions in ribosome rescue. Instead of recognizing stop codons, Pelota's primary role is to recognize and trigger the disassembly of ribosomes stalled on aberrant mRNAs [83, 84]. Pelota partners with Hbs1L (Hbs1 in yeast), a GTPase paralog of eRF3. The PELO:HBS1L complex recognizes stalled ribosomes, mirroring the eRF1‐eRF3 complex [83, 84]. However, unlike canonical termination, the PELO:HBS1L complex alone cannot resolve the stall; it requires the essential collaboration of the universal ribosome splitting factor, Rli1/ABCE1 [83, 84]. For collided ribosomes, the E3 ubiquitin ligase ZNF598 and the scaffold protein Rack1 recognize the distinct 40S‐40S interface, promoting ubiquitination of specific 40S subunit proteins [85, 86].

- Disassembly for stalled or collided ribosomes:

The primary difference between Pelo:Hbs1 and ASCC complex lies in their specific functions in RQC. In mammals, the ASCC (ASC‐1 complex), containing the ASCC3 helicase, disassembles collided ribosomes, a process requiring ZNF598 for 40S ubiquitination. This contrasts with the Pelo‐Hbs1L rescue complex [87]. In *Drosophila*, Pelo:Hbs1 is involved in mRNA surveillance and ribosome rescue, particularly addressing no‐go stalls, indicating an evolutionary divergence in RQC handling of collided ribosomes [83].

- Modification of nascent peptide chains:

The 60S ribosomal subunit, remaining associated with the incomplete nascent peptide and tRNA, recruits the RQC complex, which includes NEMF (Rqc2 in yeast, RqcH in bacteria) and Listerin (LTN1 in humans, Ltn1 in yeast) [88]. Rqc2/NEMF catalyzes the addition of C‐terminal alanine and threonine (CAT‐) tails to the nascent peptide. This modification exposes lysine residues that are then recognized by Ltn1/LTN1 for ubiquitination, potentially also serving as a degron for extra‐ribosomal degradation [89, 90, 91].

- Listerin ubiquitination and peptide degradation:

Listerin, an E3 ubiquitin ligase, ubiquitinates the nascent peptide, marking it for proteasomal degradation. This step is critical for eliminating aberrant proteins, preventing accumulation and cellular toxicity [74]. Subsequent to ubiquitination, Vms1/ANKZF1 acts as a peptidyl‐tRNA hydrolase to release the product, while Cdc48/p97 then extracts the nascent peptide chain from the 60S subunit for proteasomal degradation [92].

- 40S ribosomal subunit recycling:

The recycling of the 40S subunit in RQC differs from canonical termination. The absence of increased Tma20/MCT‐1, Tma22/DENR, and Tma64/eIF2D at the Dom34/PELO rescue site indicates distinct pathways. It remains uncertain whether 40S recycling necessitates particular molecular events following the non‐collision‐induced RQC process involving PELO‐HBS1‐ABCE1. In collision‐induced RQC, the USP10‐G3BP1 complex is recruited, deubiquitinating RPS2, RPS3, and RPS10 to prevent degradation of the modified 40S subunit [93].

Altogether, these elements work in concert to ensure the efficient clearance of stalled ribosomes and translation products.

## 4 Becoming the Center of the Vertex: Pathways That Interact With Ribosome Recycling

Translation is the sole way to convert genetic information from DNA into essential proteins. This energy‐intensive process, consuming approximately one‐fifth of cellular energy output, is highly sensitive to cellular metabolism, particularly energy dynamics [94]. This metabolic feedback is pivotal for cellular stress responses [12], and its dysregulation can lead to serious human diseases [95]. Among the stages of translation, the biological ramifications of improper ribosome disassembly are particularly significant. Intuitively, faulty ribosome recycling leads to stalling, which can obstruct protein synthesis and result in the accumulation of aberrant translation products, thereby activating stress response pathways such as the unfolded protein response (UPR) and the integrated stress response (ISR) [96]. Despite the clear implications of compromised ribosome recycling, direct evidence of human disease linked to ABCE1 mutations is currently absent from databases like OMIM (*601213) and the International Mouse Phenotyping Consortium (*Abce1*|MGI:1195458). It is plausible that mutations directly affecting ribosome recycling genes induce defects too severe to manifest as observable phenotypes. The example of PELO supports this, where the absence of direct disease‐related mutations is noted, yet its disruption causes early embryonic lethality and cell cycle abnormalities [97].

An alternative hypothesis posits the need for more precise genomic analyses to fully delineate the correlation between ABCE1 and PELO alterations and human pathologies. Emerging evidence suggests ABCE1 and PELO activity may contribute to the pathogenesis of complex, multifactorial diseases. For instance, Hbs1L deficiency, which affects Pelo levels and ribosome recycling, is associated with congenital anomalies and developmental delays, though this is linked to HBS1L mutations rather than PELO itself [98, 99]. Studies indicate that inefficient ribosome recycling can impair protein synthesis and potentially contribute to neurodegenerative conditions. Issues during elongation or termination can induce RQC, potentially disrupting protein homeostasis observed in diseases such as Alzheimer's disease [100], Parkinson's disease [78, 101], and amyotrophic lateral sclerosis (ALS) [102]. Furthermore, a study revealed that aged D1 spiny projection neurons in mouse striatum and aged human neurons displayed an accumulation of 3’ UTR mRNA fragments, correlating with reduced ABCE1 activity and suggesting a potential role in age‐related neurodegeneration [30]. ABCE1 has also been implicated in cancer progression, particularly in lung and breast cancer, where its overexpression correlates with tumor growth and metastasis [103, 104]. The Fe‐S clusters within ABCE1 are susceptible to oxidative damage, potentially explaining how oxidative stress modulates ribosome recycling efficiency within cells. These observations highlight the importance of recycling factor expression levels rather than solely functional mutations.

Ribosome stalling/collisions are thoroughly analyzed. Recent research has demonstrated that translation stalling can trigger translation inhibition via the EDF1‐GIGYF2‐EIF4E2 feedback loop [105] and induce stress responses through the ZAKα/p38/JNK [106], GCN2/1/20 [107], SAPK (p38/JNK) [75], and cGAS‐STING pathways [108]. Additionally, the inability to effectively resolve ribosome collisions can lead to ribotoxic stress responses, affecting the AMPK/mTORC1 signaling pathway [109]. These profound cellular consequences of compromised ribosome recycling may also originate from the recycling processes of 40S and 60S subunits within the RQC process. However, the discussion in this section is inadequate.

### 4.1 Signaling Pathways That Intersect With the 40S Subunit Recycling

During the RQC process, 40S ribosomal subunit proteins undergo reversible monoubiquitinated by two E3 ubiquitin ligases, ZNF598 and RNF10 [110]. Failure to deubiquitinate the marked 40S subunit by the G3BP1‐family‐USP10 complex leads to lysosomal degradation, impacting ribosomal subunit stoichiometry [93]. Our recent research highlights the 40S ribosomal subunit recycling (USP10‐G3BP1) complex's role in regulating mitochondrial dynamics and function, particularly through interactions with mitochondrial fission and fusion factors [12]. The USP10‐G3BP1 complex is also critical for the functional assembly of endoplasmic reticulum‐mitochondrial contact sites (ERMCS), which are vital for mitochondrial function and cellular energy homeostasis. Malfunctions in this process result in widespread mitochondrial dysfunction, evident through disrupted mitochondrial calcium homeostasis, mitochondrial fragmentation, and impaired oxidative phosphorylation [12]. Furthermore, the USP10‐G3BP1 complex modulates the mTORC1/2 pathway activity, suggesting a link between cellular quality control and energy fluctuations. Effective communication is crucial for alleviating proteostasis‐related stress. Deficiencies in ribosome recycling can induce mitochondrial stress, a prevalent occurrence in neurodegenerative disorders like Parkinson's and Alzheimer's disease. These findings echo the previous discovery of the EDF1‐GIGYF2‐EIF4E2 translation inhibition feedback mechanism, elucidating a prompt response to adjust energy metabolism.

Notably, FMR1 is recruited by the USP10‐G3BP1 complex in this process [12]. Previous work showed that FMRP's C‐terminal domain (FMRP‐C) interacts with voltage‐dependent anion channels (VDAC) to modulate the endoplasmic reticulum (ER)‐mitochondrial contact points (ERMCS) formation and function [111]. This suggests that RQC or 40S subunit recycling may contribute to the development of Fragile X syndrome. Collectively, these findings indicate that 40S ribosome recycling is a vital hub for cellular quality control, energy metabolism, and organelle communication, rather than merely as a passive recovery mechanism. The USP10‐G3BP1 complex is important in maintaining the mitochondrial fission–fusion balance and ER‐mitochondrial contact site stability, thereby linking ribosome recycling to a broader metabolic and stress response network. Impaired 40S ribosome recycling is now recognized as a contributor to mitochondrial stress, neurodegeneration, cancer progression, and immune system activation. The significant implications of ribosome recycling dysfunction are underscored by dysregulated mTORC1/2 signaling, misassembled stress granules, and perturbations in the cGAS‐STING pathway [112, 113, 114].

USP10 and G3BP1 are multifunctional proteins and their mutations cause human diseases not always associated with the 40S subunit recycling pathway. For example, USP10 stabilizes tau protein, leading to neurofibrillary tangles and neuronal toxicity in Alzheimer's disease (AD) [115]. USP10 mutations contribute to p53 instability in lung and breast cancers, while its overexpression in hepatocellular carcinoma stabilizes oncogenic factors such as c‐Myc, promoting tumor growth [116, 117, 118]. G3BP1 mutations enhance translational control under stress and promote tumor survival [119]. However, certain effects may be linked to the 40S subunit recycling pathway. In amyotrophic lateral sclerosis (ALS) and frontotemporal dementia (FTD), USP10 dysfunction hinders autophagy, causing misfolded protein accumulation in motor neurons [120, 121]. G3BP1 mutations induce mislocalization of key ALS‐associated proteins, TDP‐43 and FUS, by impairing stress granules formation [120, 121]. In fragile X syndrome (FXS) and autism spectrum disorder (ASD), both USP10 and G3BP1 regulate neuronal translation through FMRP interaction, and their mutations disrupt synaptic protein synthesis, impairing cognitive function and neurodevelopment [122, 123].

### 4.2 Signaling Pathways That Intersect With the 60S Subunit Recycling

The discussions of 60S subunit recycling necessitate considering the RQC/CAT‐tailing mechanism and the profound effects of its products on cellular metabolism. The composition and exact physiological role of the CAT‐tails remain incompletely understood. Amino acid composition of CAT‐tails on nascent peptide chains significantly influences their outcome. Initial studies indicate that C‐terminal polyalanine or alanine‐rich tails can function as degrons, facilitating the degradation of aberrant nascent peptide chains [124, 125]. Conversely, C‐terminal polythreonine or threonine‐rich tails demonstrate high stability and resistance to detergent solubilization [126, 127]. Polythreonine aggregation can act as seeds for the entanglement and aggregation of other species with low threonine content, sequester molecular chaperones, disrupt protein homeostasis, and trigger a heat shock response [128, 129]. The regulation of stalled nascent peptide chain composition and fate may be influenced by mechanochemical forces between them and the translation machinery, although it is not fully understood [130].

CAT‐tails, with their ability to form insoluble aggregates, have been shown to induce proteostasis stress and subsequent cell or tissue toxicity in cultured human neurons [131], *Drosophila* disease models [78], and yeast [128, 129]. A particular area of interest lies in determining the physiological functions associated with shorter and less stable CAT‐tails. Our recent study highlighted the significance of mitochondrial stress‐induced protein carboxyl‐terminal alanine and threonine tailing (msiCAT‐tailing) in glioblastoma [11]. The accumulation of CAT‐tailed mitochondrial ATP synthase F1 subunit α (ATP5α) has been shown to increase mitochondrial membrane potential and suppress the opening of mitochondrial permeability transition pore (MPTP), ultimately supporting the survival and migration of glioblastoma stem cells [11]. These findings hint that mitochondrial proteins with CAT‐tails can interfere with mitochondrial proteostasis, and that certain short tails may also possess distinctive biological roles, including promoting conditions conducive to tumor survival and chemotherapy resistance.

Although preliminary, these studies suggest that CAT‐tailing may significantly impact neurological diseases sensitive to proteostasis, and cancers experiencing high translational stress. For example, *LISTERIN* (*LTN1*) mutations may lead to early‐onset and progressive neurological and motor impairment as well as neurodegeneration [132]. *NEMF* mutations have been implicated in the onset of progressive motor neuron degeneration in mice and have been identified in patients with juvenile neuromuscular diseases [133]. Whether these phenotypes resulting from mutations in these RQC factors converge on the RQC mechanism or have distinct reasons, and what the principal protein substrates targeted by RQC in this process are, will require further investigation in subsequent research.

The involvement of RQC recycling factors in cancer pathogenesis presents a complex and intricate relationship. Initial omics‐based analyses have shown dysregulation in the expression of several RQC genes, such as *ASCC3*, *ABCE1*, *ANKZF1*, and *VCP*, in cancer cells [104, 134, 135, 136]; however, there is a lack of direct mechanistic studies. Additionally, various RQC factors (sometimes even the same factor) may demonstrate contrasting roles in promoting tumorigenesis and impeding tumor growth depending on the specific conditions. The inhibition of *ABCE1*, *ASCC3*, and *VCP* has been shown to impede cancer cell proliferation and viability [104, 134, 136], while inhibiting *NEMF*/*Clbn* and *ZNF598* may facilitate cancer cell growth and survival [137, 138]. Recent research showcased the involvement of ANKZF1 in maintaining mitochondrial proteostasis and its influence on glioblastoma progression [139]. Nevertheless, this research used a nonphysiological mitochondrial‐targeted GFP to induce proteostasis stress in the matrix, and the role of endogenous CAT‐tail‐modified mitochondrial proteins in this process is not clearly understood. This suggests that the functions of RQC factors in cancer cells are intricately nuanced and highly influenced by both genetic and environmental contexts. The molecular mechanisms underlying the distinct effects of RQC genes in cancer biology present an intriguing area for prospective investigation.

Furthermore, while our current comprehension of mitochondrial ribosome quality control remains limited, the pivotal components mtRF‐R and MTRES1 are demonstrably crucial for mitochondrial protein synthesis. Deletion of *MTRES1* and *mtRF‐R* in human cells results in decreased mitochondrial translation and impairments in oxidative phosphorylation [140, 141]. Variations in *mtRF‐R* potentially cause a combination of optic atrophy, peripheral neuropathy, and spastic paraplegia in human neurological diseases such as Leigh syndrome, indicating the essential involvement of mitochondrial function in neurons [142, 143, 144].

## 5 Finale: Tracing Ribosome Recycling Imbalance in Human Disease

**FIGURE 1:**
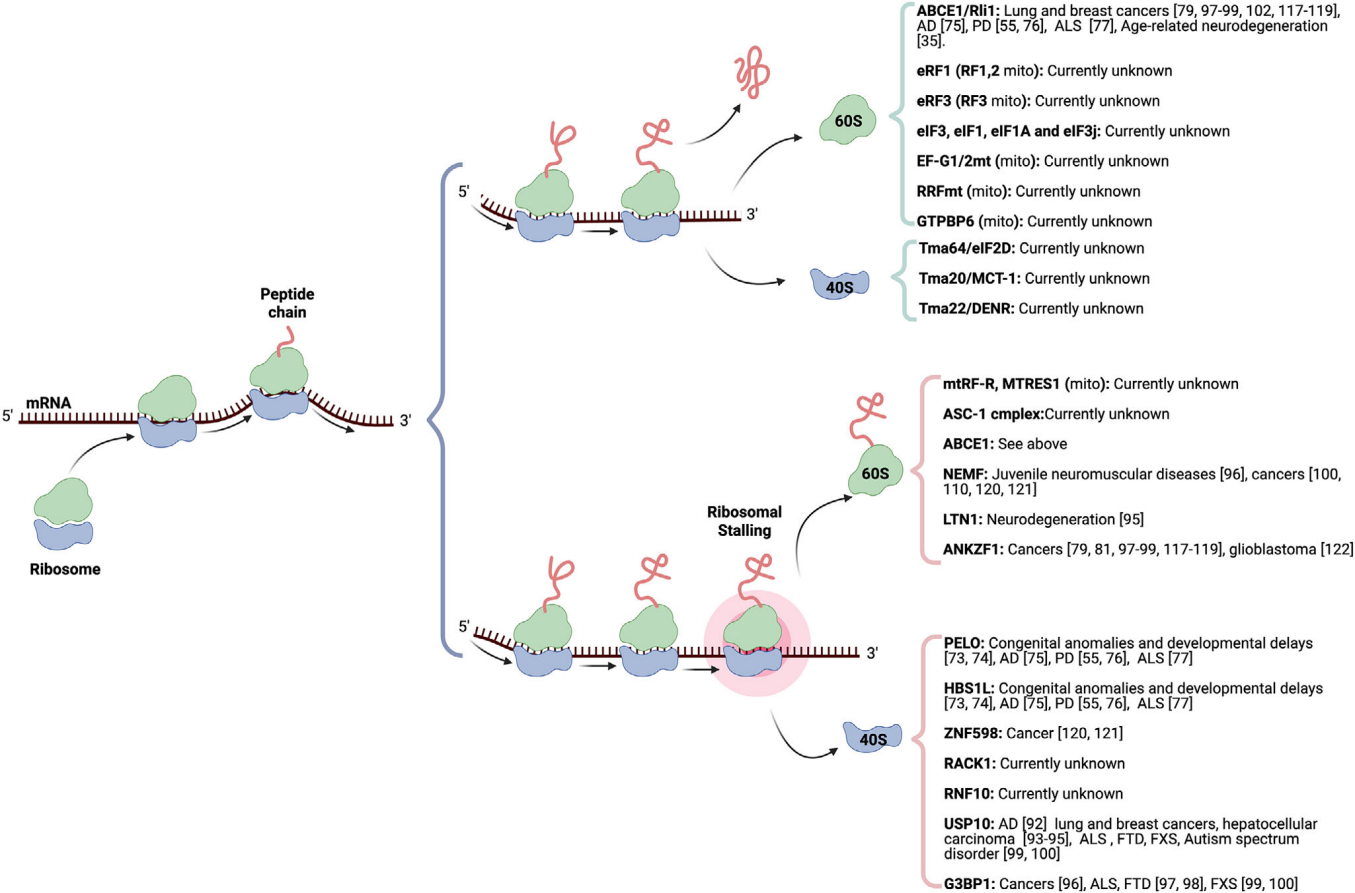
Mechanisms of ribosome recycling and their relevance to disease. This schematic illustrates translation and the cellular response to ribosomal stalling. Under normal conditions (top), ribosomes translate mRNA from the 5’ to the 3’ end, synthesize peptide chains, and then disassemble and recycle the 60S and 40S ribosomal subunits. In ribosome stalling (bottom), collided ribosomes are recognized by the ribosome‐associated quality control (RQC) machinery, leading to the dissociation of the subunits and degradation of the nascent peptide. Key proteins involved in RQC and ribosome recycling are shown to the right, along with associated diseases, including neurodegenerative diseases, cancer, autism spectrum disorder, and developmental delays. Proteins such as ABCE1, USP10, G3BP1, and ANKZF1 are implicated in multiple disease pathways, underscoring the critical role of translational quality control in human health.

This succinct overview summarizes the ribosome recycling process and its links to human pathologies (Figure 1). The investigation is challenging due to limited and fragmented evidence. Eukaryotic cells employ distinct molecular mechanisms for routine translation termination and unexpected translational arrest. For example, the 40S subunit recycling factors Tma64/eIF2D, Tma20/MCT‐1, and Tma22/DENR are not utilized in RQC, and the USP10‐G3BP1 complex is absent in canonical recycling. The evolutionary rationale behind these differences remains an open question. A major hurdle in studying ribosome recycling defects in human disease is the scarcity of direct genetic evidence. Many mutations in ribosome‐associated pathways often lead to severe developmental defects or early lethality, hindering their detection in human genetic analyses. Current detection techniques also pose limitations, particularly in resolving subtle traces of ribosome recycling disorders during disease assessment. These limitations underscore the need for novel detection approaches that can reveal the nuanced, long‐term consequences of ribosome recycling defects. Advanced genetic screening methods, such as expression quantitative trait loci (eQTL) analysis and high‐resolution proteomic profiling, show promise for identifying disease‐associated variations in ribosome recycling factors [145, 146, 147]. The narrative of *ABCE1* suggests that single‐nucleotide polymorphisms (SNPs) associated with the ribosome recycling machinery may regulate the expression of these factors.

Of interest is where to look for clues about ribosome recycling imbalance. Protein synthesis is a cyclical process universally divided into the following four basic phases: initiation, elongation, termination, and ribosome recycling. Each phase is vital for maintaining translation fidelity and efficiency, and dysregulation can directly or indirectly impact subsequent ribosome cycling. While previous work focused on ribosome cycling after elongation and termination, the initiation phase warrants particular attention. It is the most complex and highly regulated step, serving as the rate‐limiting point that dictates the overall protein production efficiency for a given mRNA [148]. Therefore, understanding the connection between translation initiation and quality control mechanisms is especially compelling.

Eukaryotic cells have evolved a predominant, canonical initiation pathway dependent on a 5’ cap structure. Additionally, diverse non‐canonical pathways offer regulatory flexibility during cellular stress, when eIF4E‐dependent translation is inhibited. They included internal translations mediated by the internal ribosome entry sites (IRESs), cap‐independent translational enhancers (CITEs), and N6‐methyladenosine (m^6^A) modification [149, 150]. Alternative initiation factors like DAP5 and translation initiation factors of short 5’ UTRs (TISU) also facilitate scanless translation of mRNAs with very short leaders [149, 150]. Furthermore, Repeat‐Associated Non‐AUG (RAN) translation can initiate from expanded, GC‐rich nucleotide repeats, as found in neurodegenerative diseases, without the canonical AUG start codon [151, 152]. These diverse mechanisms reveal a profound duality in non‐canonical translation. On one hand, pathways such as IRES‐ and m^6^A‐mediated initiation represent elegant adaptive strategies, enabling cells to bypass the highly regulated canonical pathway to produce vital proteins during crises when the main system is shut down. This circumvention of standard checkpoints is a pro‐survival tactic. On the other hand, this same principle of bypassing canonical fidelity checks can be exploited in disease [149, 153]. Inappropriate ribosome loading during this phase can lead to translation imbalance, triggering quality control mechanisms to restore homeostasis.

The precise detection of RQC/CAT‐tailing products offers valuable applications in disease monitoring, particularly in cancers and neurodegenerative diseases discussed previously. However, developing detection antibodies for identifying CAT‐tailed nascent peptide chains remains challenging due to the low antigenicity of alanine and threonine, as well as the heterogeneity of tail species [78, 126, 127]. Future investigations into the amino acid composition and primary sequence of CAT‐tails will facilitate this goal. A thorough examination of ribosome recycling's role in disease onset may provide new insights into translation control and proteostasis maintenance under healthy conditions. Furthermore, this understanding can be leveraged to combat prevalent diseases. Moving forward, further investigations into the intersection of ribosome recycling, mitochondrial quality control, and immune regulation may reveal critical links between translation dysfunction and disease pathology. As detection technologies advance, we approach the leveraging of these insights for precision medicine, unlocking novel opportunities for early disease diagnosis, biomarker discovery, and targeted therapeutic interventions.

## 6 Conclusion

Ribosome recycling, previously considered a fundamental translation process, is now recognized as a critical regulator of cellular quality control, organelle function, and disease pathogenesis. While direct links between genetic mutations in core recycling factors (e.g., ABCE1, PELO) and human diseases are infrequent, dysregulation of these factors indirectly contributes to various pathological conditions. Ineffective ribosome recycling leads to ribosomal stalling, stress signaling, and proteostasis disruption, culminating in organelle dysfunction, particularly in mitochondria. These disruptions are implicated in neurodegenerative diseases, developmental disorders, and cancers. This review highlights two distinct recycling branches: 40S subunit recycling, mediated by the USP10‐G3BP1 complex and linked to mitochondrial dynamics, and 60S subunit disassembly via RQC/CAT‐tailing, influencing cellular stress, protein aggregation, and tumor progression. The dual nature of CAT‐tailing proteins, both toxic and adaptive, underscores the intricate balance of translational quality control in disease. Furthermore, emerging evidence indicates that ribosome recycling intersects with key signaling pathways, including GCN2, mTORC1/2, and cGAS‐STING, positioning it as a central cellular signaling hub. In conclusion, ribosome recycling is a pivotal nexus connecting translation, organelle health, and disease. Elucidating its mechanisms and signaling crosstalk offers promising avenues for identifying novel therapeutic targets to address translational stress and organelle failure, ultimately restoring proteostasis and cellular equilibrium in disease states.

## Author Contributions

F.T. and Z.W. conceived the outline. F.T. wrote the first draft of the manuscript. F.T. and Z.W. wrote and revised the manuscript.

## Conflicts of Interest

The authors declare no conflicts of interest.

## Data Availability

Data sharing does not apply to this article, as no datasets were generated or analyzed during the current study.

## References

[1] D. J. Young and N. R. Guydosh , “Rebirth of the Translational Machinery: The Importance of Recycling Ribosomes,” BioEssays: News and Reviews in Molecular, Cellular and Developmental Biology 44, no. 4 (2022): e2100269, 10.1002/bies.202100269. PMC9270684 35147231

[2] E. Nürenberg‐Goloub and R. Tampé , “Ribosome Recycling in mRNA Translation, Quality Control, and Homeostasis,” Biological Chemistry 401, no. 1 (2019): 47–61, 10.1515/hsz-2019-0279. 31665102

[3] J. Brito Querido , I. Díaz‐López , and V. Ramakrishnan , “The Molecular Basis of Translation Initiation and Its Regulation in Eukaryotes,” Nature Reviews Molecular Cell Biology 25, no. 3 (2024): 168–186, 10.1038/s41580-023-00624-9. 38052923

[4] C. U. T. Hellen , “Translation Termination and Ribosome Recycling in Eukaryotes,” Cold Spring Harbor Perspectives in Biology 10, no. 10 (2018): a032656, 10.1101/cshperspect.a032656. 29735640 PMC6169810

[5] F. Nadler , E. Lavdovskaia , and R. Richter‐Dennerlein , “Maintaining Mitochondrial Ribosome Function: The Role of Ribosome Rescue and Recycling Factors,” RNA Biology 19, no. 1 (2022): 117–131, 10.1080/15476286.2021.2015561. 34923906 PMC8786322

[6] K. N. D'Orazio and R. Green , “Ribosome States Signal RNA Quality Control,” Molecular Cell 81, no. 7 (2021): 1372–1383, 10.1016/j.molcel.2021.02.022. 33713598 PMC8041214

[7] S. Meydan and N. R. Guydosh , “A Cellular Handbook for Collided Ribosomes: Surveillance Pathways and Collision Types,” Current Genetics 67, no. 1 (2021): 19–26, 10.1007/s00294-020-01111-w. 33044589 PMC7887001

[8] J. R. Warner , “The Economics of Ribosome Biosynthesis in Yeast,” Trends in Biochemical Sciences 24, no. 11 (1999): 437–440, 10.1016/s0968-0004(99)01460-7. 10542411

[9] S. Filbeck , F. Cerullo , S. Pfeffer , and C. A. P. Joazeiro , “Ribosome‐associated Quality‐Control Mechanisms From Bacteria to Humans,” Molecular Cell 82, no. 8 (2022): 1451–1466, 10.1016/j.molcel.2022.03.038. 35452614 PMC9034055

[10] J. Geng , S. Li , Y. Li , et al., “Stalled Translation by Mitochondrial Stress Upregulates a CNOT4‐ZNF598 Ribosomal Quality Control Pathway Important for Tissue Homeostasis,” Nature Communications 15, no. 1 (2024): 1637, 10.1038/s41467-024-45525-3. PMC10883933 38388640

[11] T. Cai , B. Zhang , E. Reddy , et al., “The Mitochondrial Stress‐Induced Protein Carboxyl‐Terminal Alanine and Threonine Tailing (msiCAT‐Tailing) Promotes Glioblastoma Tumorigenesis by Modulating Mitochondrial Functions,” eLife 13 (2024): RP99438, 10.7554/eLife.99438.1. PMC12854676 41609019

[12] F. Tahmasebinia , Y. Tang , R. Tang , et al., “The 40S Ribosomal Subunit Recycling Complex Modulates Mitochondrial Dynamics and Endoplasmic Reticulum—Mitochondria Tethering at Mitochondrial Fission/Fusion Hotspots,” Nature Communications 16, no. 1 (2025): 1021, 10.1038/s41467-025-56346-3. PMC11762756 39863576

[13] B. Lu , “Translational Regulation by Ribosome‐Associated Quality Control in Neurodegenerative Disease, Cancer, and Viral infection,” Frontiers in Cell and Developmental Biology 10 (2022): 970654, 10.3389/fcell.2022.970654. 36187485 PMC9515510

[14] A. Biever , C. Glock , G. Tushev , et al., “Monosomes Actively Translate Synaptic mRNAs in Neuronal Processes,” Science 367 (2020): aay4991, 10.1126/science.aay4991. 32001627

[15] M. Kapur , C. E. Monaghan , and S. L. Ackerman , “Regulation of mRNA Translation in Neurons‐A Matter of Life and Death,” Neuron 96, no. 3 (2017): 616–637, 10.1016/j.neuron.2017.09.057. 29096076 PMC5693308

[16] J. Wang , J. Wang , H. Cao , et al., “The Relationship Between Ribosome‐Associated Quality Control and Neurological Disorders,” Series A, Biological Sciences and Medical Sciences 80, no. 4 (2025): glae304, 10.1093/gerona/glae304. 39719885

[17] N. Robichaud , N. Sonenberg , D. Ruggero , and R. J. Schneider , “Translational Control in Cancer,” Cold Spring Harbor Perspectives in Biology 11, no. 7 (2019): a032896, 10.1101/cshperspect.a032896. 29959193 PMC6601465

[18] C. Ni and M. Buszczak , “The Homeostatic Regulation of Ribosome biogenesis,” Seminars in Cell & Developmental Biology 136 (2023): 13–26, 10.1016/j.semcdb.2022.03.043. 35440410 PMC9569395

[19] M. S. Savannah , “Mechanisms of Ribosome Recycling in Bacteria and Mitochondria: A Structural Perspective,” RNA Biology 19, no. 1 (2025): 662, 10.1080/15476286.2022.2067712. PMC9067457 35485608

[20] M. W. Gray , G. Burger , and B. F. Lang , “Mitochondrial Evolution,” Science 283, no. 5407 (1999): 1476–1481, 10.1126/science.283.5407.1476. 10066161

[21] C. Trahan and M. Oeffinger , “The Importance of Being RNA‐est: Considering RNA‐Mediated Ribosome Plasticity,” RNA Biology 20, no. 1 (2023): 177–185, 10.1080/15476286.2023.2204581. 37098839 PMC10134959

[22] K. Dörner , C. Ruggeri , I. Zemp , and U. Kutay , “Ribosome Biogenesis Factors‐From Names to Functions,” EMBO Journal 42, no. 7 (2023): 112699, 10.15252/embj.2022112699. PMC10068337 36762427

[23] R. K. Koripella , M. R. Sharma , P. Risteff , P. Keshavan , and R. K. Agrawal , “Structural Insights Into Unique Features of the human Mitochondrial Ribosome Recycling,” Proceedings of the National Academy of Sciences of the United States of America 116, no. 17 (2019): 8283–8288, 10.1073/pnas.1815675116. 30962385 PMC6486771

[24] E. O. van der Sluis , H. Bauerschmitt , T. Becker , et al., “Parallel Structural Evolution of Mitochondrial Ribosomes and OXPHOS Complexes,” Genome Biology and Evolution 7, no. 5 (2015): 1235–1251, 10.1093/gbe/evv061. 25861818 PMC4453056

[25] V. Skaltsogiannis , T.‐T. Nguyen , N. Corre , et al., “Structural Insights Into Maturation and Translation of a Plant Mitoribosome,” BioRxiv (2024), 10.1101/2024.10.28.620559.

[26] S. A. Ayyub and U. Varshney , “Translation Initiation in Mammalian Mitochondria – A Prokaryotic Perspective,” RNA Biology 17, no. 2 (2020): 165–175, 10.1080/15476286.2019.1690099. 31696767 PMC6973315

[27] Y. Inagaki and W. Ford Doolittle , “Evolution of the Eukaryotic Translation Termination System: Origins of Release Factors,” Molecular Biology and Evolution 17, no. 6 (2000): 882–889, 10.1093/oxfordjournals.molbev.a026368. 10833194

[28] A. des Georges , Y. Hashem , A. Unbehaun , et al., “Structure of the Mammalian Ribosomal Pre‐termination Complex Associated With eRF1.ERF3.GDPNP,” Nucleic Acids Research 42, no. 5 (2014): 3409–3418, 10.1093/nar/gkt1279. 24335085 PMC3950680

[29] D. J. Young , N. R. Guydosh , F. Zhang , A. G. Hinnebusch , and R. Green , “Rli1/ABCE1 Recycles Terminating Ribosomes and Controls Translation Reinitiation in 3′UTRs In Vivo,” Cell 162, no. 4 (2015): 872–884, 10.1016/j.cell.2015.07.041. 26276635 PMC4556345

[30] P. H. Sudmant , H. Lee , D. Dominguez , M. Heiman , and C. B. Burge , “Widespread Accumulation of Ribosome‐Associated Isolated 3' UTRs in Neuronal Cell Populations of the Aging Brain,” Cell Reports 25, no. 9 (2018): 2447–2456. e4, 10.1016/j.celrep.2018.10.094. 30485811 PMC6354779

[31] A. V. Pisarev , M. A. Skabkin , V. P. Pisareva , et al., “The Role of ABCE1 in Eukaryotic Posttermination Ribosomal Recycling,” Molecular Cell 37, no. 2 (2010): 196–210, 10.1016/j.molcel.2009.12.034. 20122402 PMC2951834

[32] C. J. Shoemaker and R. Green , “Kinetic Analysis Reveals the Ordered Coupling of Translation Termination and Ribosome Recycling in Yeast,” Proceedings of the National Academy of Sciences of the United States of America 108, no. 51 (2011): E1392–E1398, 10.1073/pnas.1113956108. 22143755 PMC3251084

[33] A. Brown , S. Shao , J. Murray , R. S. Hegde , and V. Ramakrishnan , “Structural Basis for Stop Codon Recognition in Eukaryotes,” Nature 524, no. 7566 (2015): 493–496, 10.1038/nature14896. 26245381 PMC4591471

[34] T. Becker , S. Franckenberg , S. Wickles , et al., “Structural Basis of Highly Conserved Ribosome Recycling in Eukaryotes and Archaea,” Nature 482, no. 7386 (2012): 501–506, 10.1038/nature10829. 22358840 PMC6878762

[35] A. Karcher , K. Büttner , B. Märtens , R. P. Jansen , and K. P. Hopfner , “X‐ray Structure of RLI, an Essential Twin Cassette ABC ATPase Involved in Ribosome Biogenesis and HIV Capsid Assembly,” Structure (London, England) 13, no. 4 (2005): 649–659, 10.1016/j.str.2005.02.008. 15837203

[36] D. Barthelme , S. Dinkelaker , S. V. Albers , P. Londei , U. Ermler , and R. Tampé , “Ribosome Recycling Depends on a Mechanistic Link Between the FeS Cluster Domain and a Conformational Switch of the Twin‐ATPase ABCE1,” Proceedings of the National Academy of Sciences of the United States of America 108, no. 8 (2011): 3228–3233, 10.1073/pnas.1015953108. 21292982 PMC3044390

[37] G. Gouridis , B. Hetzert , K. Kiosze‐Becker , et al., “ABCE1 Controls Ribosome Recycling by an Asymmetric Dynamic Conformational Equilibrium,” Cell Reports 28, no. 3 (2019): 723–734. e6, 10.1016/j.celrep.2019.06.052. 31315050 PMC6656783

[38] J. Brito Querido , M. Sokabe , S. Kraatz , et al., “Structure of a Human 48 S Translational Initiation Complex,” Science 369, no. 6508 (2020): 1220–1227, 10.1126/science.aba4904. 32883864 PMC7116333

[39] H. Kratzat , T. Mackens‐Kiani , M. Ameismeier , et al., “A Structural Inventory of Native Ribosomal ABCE1‐43S Pre‐Initiation Complexes,” EMBO Journal 40, no. 1 (2021): 105179, 10.15252/embj.2020105179. PMC7780240 33289941

[40] M. A. Skabkin , O. V. Skabkina , V. Dhote , A. A. Komar , C. U. Hellen , and T. V. Pestova , “Activities of Ligatin and MCT‐1/DENR in Eukaryotic Translation Initiation and Ribosomal Recycling,” Genes & Development 24, no. 16 (2010): 1787–1801, 10.1101/gad.1957510. 20713520 PMC2922506

[41] T. C. Fleischer , C. M. Weaver , K. J. McAfee , J. L. Jennings , and A. J. Link , “Systematic Identification and Functional Screens of Uncharacterized Proteins Associated With Eukaryotic Ribosomal Complexes,” Genes & Development 20, no. 10 (2006): 1294–1307, 10.1101/gad.1422006. 16702403 PMC1472904

[42] D. J. Young , D. S. Makeeva , F. Zhang , et al., “Tma64/eIF2D, Tma20/MCT‐1, and Tma22/DENR Recycle Post‐Termination 40S Subunits In Vivo,” Molecular Cell 71, no. 5 (2018): 761–774. e5, 10.1016/j.molcel.2018.07.028. 30146315 PMC6225905

[43] J. Bohlen , L. Harbrecht , S. Blanco , et al., “DENR Promotes Translation Reinitiation via Ribosome Recycling to Drive Expression of Oncogenes Including ATF4,” Nature Communications 11, no. 1 (2020): 4676, 10.1038/s41467-020-18452-2. PMC7494916 32938922

[44] D. J. Young , S. Meydan , and N. R. Guydosh , “40S ribosome Profiling Reveals Distinct Roles for Tma20/Tma22 (MCT‐1/DENR) and Tma64 (eIF2D) in 40S Subunit Recycling,” Nature Communications 12, no. 1 (2021): 2976, 10.1038/s41467-021-23223-8. PMC8137927 34016977

[45] A. V. Pisarev , C. U. Hellen , and T. V. Pestova , “Recycling of Eukaryotic Posttermination Ribosomal Complexes,” Cell 131, no. 2 (2007): 286–299, 10.1016/j.cell.2007.08.041. 17956730 PMC2651563

[46] R. Meurs , M. De Matos , A. Bothe , et al., “MCTS2 and Distinct eIF2D Roles in uORF‐Dependent Translation Regulation Revealed by In Vitro Re‐Initiation Assays,” EMBO Journal 44, no. 3 (2025): 854–876, 10.1038/s44318-024-00347-3. 39748120 PMC11790910

[47] M. Laurberg , H. Asahara , A. Korostelev , J. Zhu , S. Trakhanov , and H. F. Noller , “Structural Basis for Translation Termination on the 70S Ribosome,” Nature 454, no. 7206 (2008): 852–857, 10.1038/nature07115. 18596689

[48] S. Petry , D. E. Brodersen , F. V. Murphy , et al., “Crystal Structures of the Ribosome in Complex With Release Factors RF1 and RF2 Bound to a Cognate Stop Codon,” Cell 123, no. 7 (2005): 1255–1266, 10.1016/j.cell.2005.09.039. 16377566

[49] Z. Fu , G. Indrisiunaite , S. Kaledhonkar , et al., “The Structural Basis for Release‐Factor Activation During Translation Termination Revealed by Time‐Resolved Cryogenic Electron Microscopy,” Nature Communications 10, no. 1 (2019): 2579, 10.1038/s41467-019-10608-z. PMC6561943 31189921

[50] F. Nadler and R. Richter‐Dennerlein , “Translation Termination in Human Mitochondria—Substrate Specificity of Mitochondrial Release Factors,” Biological Chemistry 404 (2023): 9, 10.1515/hsz-2023-0127. 37377370

[51] M. Saurer , M. Leibundgut , H. P. Nadimpalli , et al., “Molecular Basis of Translation Termination at Noncanonical Stop Codons in human Mitochondria,” Science 380, no. 6644 (2023): 531–536, 10.1126/science.adf9890. 37141370

[52] J. Pallesen , Y. Hashem , G. Korkmaz , et al., “Cryo‐EM Visualization of the Ribosome in Termination Complex With Apo‐RF3 and RF1,” Elife 2 (2013): 00411, 10.7554/eLife.00411. PMC3677378 23755360

[53] Z. M. Chrzanowska‐Lightowlers and R. N. Lightowlers , “Translation in Mitochondrial Ribosomes,” Methods in Molecular Biology 2661 (2023): 53–72, 10.1007/978-1-0716-3171-3_4. 37166631

[54] Z. Fu , S. Kaledhonkar , A. Borg , et al., “Key Intermediates in Ribosome Recycling Visualized by Time‐Resolved Cryoelectron Microscopy,” Structure (London, England) 24, no. 12 (2016): 2092–2101, 10.1016/j.str.2016.09.014. PMC5143168 27818103

[55] R. D. Pai , W. Zhang , B. S. Schuwirth , et al., “Structural Insights Into Ribosome Recycling Factor Interactions With the 70S Ribosome,” Journal of Molecular Biology 376, no. 5 (2008): 1334–1347, 10.1016/j.jmb.2007.12.048. 18234219 PMC2712656

[56] A. Weixlbaumer , S. Petry , C. M. Dunham , M. Selmer , A. C. Kelley , and V. Ramakrishnan , “Crystal Structure of the Ribosome Recycling Factor Bound to the Ribosome,” Nature Structural & Molecular Biology 14, no. 8 (2007): 733–777, 10.1038/nsmb1282. 17660830

[57] L. Lancaster , M. C. Kiel , A. Kaji , and H. F. Noller , “Orientation of Ribosome Recycling Factor in the Ribosome From Directed Hydroxyl Radical Probing,” Cell 111, no. 1 (2002): 129–140, 10.1016/s0092-8674(02)00938-8. 12372306

[58] S. M. Seely and M. G. Gagnon , “Mechanisms of Ribosome Recycling in Bacteria and Mitochondria: A Structural Perspective,” RNA Biology 19, no. 1 (2022): 129–140, 10.1080/15476286.2022.2067712. PMC9067457 35485608

[59] H. Stark , M. V. Rodnina , H. J. Wieden , M. van Heel , and W. Wintermeyer , “Large‐Scale Movement of Elongation Factor G and Extensive Conformational Change of the Ribosome During Translocation,” Cell 100, no. 3 (2000): 301–309, 10.1016/s0092-8674(00)80666-2. 10676812

[60] A. Borg , M. Pavlov , and M. Ehrenberg , “Complete Kinetic Mechanism for Recycling of the Bacterial Ribosome,” RNA 22, no. 1 (2016): 10–21, 10.1261/rna.053157.115. 26527791 PMC4691825

[61] R. Shalgi , J. A. Hurt , I. Krykbaeva , M. Taipale , S. Lindquist , and C. B. Burge , “Widespread Regulation of Translation by Elongation Pausing in Heat Shock,” Molecular Cell 49, no. 3 (2013): 439–452, 10.1016/j.molcel.2012.11.028. 23290915 PMC3570722

[62] K. R. Hurst‐Hess , P. Rudra , and P. Ghosh , “Ribosome Protection as a Mechanism of Lincosamide Resistance in Mycobacterium abscessus,” Antimicrobial Agents and Chemotherapy 65, no. 11 (2021): 0118421, 10.1128/AAC.01184-21. PMC8522731 34460298

[63] P. Rudra , K. R. Hurst‐Hess , K. L. Cotten , A. Partida‐Miranda , and P. Ghosh , “Mycobacterial HflX Is a Ribosome Splitting Factor That Mediates Antibiotic Resistance,” Proceedings of the National Academy of Sciences of the United States of America 117, no. 1 (2020): 629–634, 10.1073/pnas.1906748117. 31871194 PMC6955381

[64] T. O. Koller , K. J. Turnbull , K. Vaitkevicius , et al., “Structural Basis for HflXr‐Mediated Antibiotic Resistance in Listeria monocytogenes,” Nucleic Acids Research 50, no. 19 (2022): 11285–11300, 10.1093/nar/gkac934. 36300626 PMC9638945

[65] Y. Zhang , C. S. Mandava , W. Cao , et al., “HflX Is a Ribosome‐Splitting Factor Rescuing Stalled Ribosomes Under Stress Conditions,” Nature Structural & Molecular Biology 22, no. 11 (2015): 906–913, 10.1038/nsmb.3103. 26458047

[66] S. M. Seely , R. S. Basu , and M. G. Gagnon , “Mechanistic Insights Into the Alternative Ribosome Recycling by HflXr,” Nucleic Acids Research 52, no. 7 (2024): 4053–4066, 10.1093/nar/gkae128. 38407413 PMC11040002

[67] M. L. Coatham , H. E. Brandon , J. J. Fischer , T. Schümmer , and H. J. Wieden , “The Conserved GTPase HflX Is a Ribosome Splitting Factor That Binds to the E‐Site of the Bacterial Ribosome,” Nucleic Acids Research 44, no. 4 (2016): 1952–1961, 10.1093/nar/gkv1524. 26733579 PMC4770234

[68] E. Lavdovskaia , K. Denks , F. Nadler , et al., “Dual Function of GTPBP6 in Biogenesis and Recycling of human Mitochondrial Ribosomes,” Nucleic Acids Research 48 (2020): 12929–12942, 10.1093/nar/gkaa1132. 33264405 PMC7736812

[69] H. S. Hillen , E. Lavdovskaia , F. Nadler , et al., “Structural Basis of GTPase‐Mediated Mitochondrial Ribosome Biogenesis and Recycling,” Nature Communications 12 (2021): 3672, 10.1038/s41467-021-23702-y. PMC8209004 34135319

[70] J. Rorbach , R. Richter , H. J. Wessels , et al., “The Human Mitochondrial Ribosome Recycling Factor Is Essential for Cell Viability,” Nucleic Acids Research 36, no. 18 (2008): 5787–5799, 10.1093/nar/gkn576. 18782833 PMC2566884

[71] S. Majumdar , A. Kashyap , R. K. Koripella , et al., “HflX‐Mediated Drug Resistance Through Ribosome Splitting and rRNA Disordering in Mycobacteria,” Proceedings of the National Academy of Sciences of the United States of America 122, no. 6 (2025): 2419826122, 10.1073/pnas.2419826122. PMC11831132 39913204

[72] M. Tsuboi , H. Morita , Y. Nozaki , et al., “EF‐G2mt Is an Exclusive Recycling Factor in Mammalian Mitochondrial Protein Synthesis,” Molecular Cell 35, no. 4 (2009): 502–510, 10.1016/j.molcel.2009.06.028. 19716793

[73] S. Ito‐Harashima , K. Kuroha , T. Tatematsu , and T. Inada , “Translation of the Poly(A) Tail Plays Crucial Roles in Nonstop mRNA Surveillance via Translation Repression and Protein Destabilization by Proteasome in Yeast,” Genes & Development 21, no. 5 (2007): 519–524, 10.1101/gad.1490207. 17344413 PMC1820893

[74] M. H. Bengtson and C. A. Joazeiro , “Role of a Ribosome‐Associated E3 Ubiquitin Ligase in Protein Quality Control,” Nature 467, no. 7314 (2010): 470–473, 10.1038/nature09371. 20835226 PMC2988496

[75] C. C. Wu , A. Peterson , B. Zinshteyn , S. Regot , and R. Green , “Ribosome Collisions Trigger General Stress Responses to Regulate Cell Fate,” Cell 182, no. 2 (2020): 404–416. e14, 10.1016/j.cell.2020.06.006. 32610081 PMC7384957

[76] Y. Harigaya and R. Parker , “No‐go Decay: A Quality Control Mechanism for RNA in Translation,” Wiley Interdisciplinary Reviews. RNA 1, no. 1 (2010): 132–141, 10.1002/wrna.17. 21956910

[77] A. R. Buskirk and R. Green , “Ribosome Pausing, Arrest and Rescue in Bacteria and Eukaryotes,” Philosophical Transactions of the Royal Society of London. Series B, Biological Sciences 372, no. 1716 (2017): 20160183, 10.1098/rstb.2016.0183. 28138069 PMC5311927

[78] Z. Wu , I. Tantray , J. Lim , et al., “MISTERMINATE Mechanistically Links Mitochondrial Dysfunction with Proteostasis Failure,” Molecular Cell 75, no. 4 (2019): 835–848. e8, 10.1016/j.molcel.2019.06.031. 31378462 PMC7362879

[79] K. Q. Kim and H. S. Zaher , “Canary in a Coal Mine: Collided Ribosomes as Sensors of Cellular Conditions,” Trends in Biochemical Sciences 47, no. 1 (2022): 82–97, 10.1016/j.tibs.2021.09.001. 34607755 PMC8688274

[80] P. Jadiya and D. Tomar , “Mitochondrial Protein Quality Control Mechanisms,” Genes 11, no. 5 (2020): 563, 10.3390/genes11050563. 32443488 PMC7290828

[81] S. A. Ayyub , F. Gao , R. N. Lightowlers , and Z. M. Chrzanowska‐Lightowlers , “Rescuing Stalled Mammalian Mitoribosomes—what Can We Learn From Bacteria?,” Journal of Cell Science 133, no. 1 (2020): jcs231811, 10.1242/jcs.231811. 31896602

[82] N. Desai , H. Yang , V. Chandrasekaran , R. Kazi , M. Minczuk , and V. Ramakrishnan , “Elongational Stalling Activates Mitoribosome‐Associated Quality Control,” Science 370, no. 6520 (2020): 1105–1110. 33243891 10.1126/science.abc7782 PMC7116630

[83] V. P. Pisareva , M. A. Skabkin , C. U. Hellen , T. V. Pestova , and A. V. Pisarev , “Dissociation by Pelota, Hbs1 and ABCE1 of Mammalian Vacant 80S Ribosomes and Stalled Elongation Complexes,” EMBO Journal 30, no. 9 (2011): 1804–1817, 10.1038/emboj.2011.93. 21448132 PMC3101999

[84] D. Taylor , A. Unbehaun , W. Li , et al., “Cryo‐EM Structure of the Mammalian Eukaryotic Release Factor eRF1‐eRF3‐associated Termination Complex,” Proceedings of the National Academy of Sciences of the United States of America 109, no. 45 (2012): 18413–18418, 10.1073/pnas.1216730109. 23091004 PMC3494903

[85] E. Sundaramoorthy , M. Leonard , R. Mak , J. Liao , A. Fulzele , and E. J. Bennett , “ZNF598 and RACK1 Regulate Mammalian Ribosome‐Associated Quality Control Function by Mediating Regulatory 40S Ribosomal Ubiquitylation,” Molecular Cell 65, no. 4 (2017): 751–760. e4, 10.1016/j.molcel.2016.12.026. 28132843 PMC5321136

[86] S. Juszkiewicz , V. Chandrasekaran , Z. Lin , S. Kraatz , V. Ramakrishnan , and R. S. Hegde , “ZNF598 Is a Quality Control Sensor of Collided Ribosomes,” Molecular Cell 72, no. 3 (2018): 469–481. e7, 10.1016/j.molcel.2018.08.037. 30293783 PMC6224477

[87] S. Juszkiewicz , S. H. Speldewinde , L. Wan , J. Q. Svejstrup , and R. S. Hegde , “The ASC‐1 Complex Disassembles Collided Ribosomes,” Molecular Cell 79, no. 4 (2020): 603–614. e8, 10.1016/j.molcel.2020.06.006. 32579943 PMC7447978

[88] C. A. P. Joazeiro , “Mechanisms and Functions of Ribosome‐associated Protein Quality Control,” Nature Reviews Molecular Cell Biology 20, no. 6 (2019): 368–383, 10.1038/s41580-019-0118-2. 30940912 PMC7138134

[89] O. Brandman , J. Stewart‐Ornstein , D. Wong , et al., “A Ribosome‐Bound Quality Control Complex Triggers Degradation of Nascent Peptides and Signals Translation Stress,” Cell 151, no. 5 (2012): 1042–1054, 10.1016/j.cell.2012.10.044. 23178123 PMC3534965

[90] P. S. Shen , J. Park , Y. Qin , et al., “Rqc2p and 60 S Ribosomal Subunits Mediate mRNA‐Independent Elongation of Nascent Chains,” Science 347, no. 6217 (2015): 75–78, 10.1126/science.1259724. 25554787 PMC4451101

[91] O. Brandman and R. S. Hegde , “Ribosome‐Associated Protein Quality Control,” Nature Structural & Molecular Biology 23, no. 1 (2016): 7–15, 10.1038/nsmb.3147. PMC4853245 26733220

[92] S. Shao , A. Brown , B. Santhanam , and R. S. Hegde , “Structure and Assembly Pathway of the Ribosome Quality Control Complex,” Molecular Cell 57, no. 3 (2015): 433–444, 10.1016/j.molcel.2014.12.015. 25578875 PMC4321881

[93] C. Meyer , A. Garzia , P. Morozov , H. Molina , and T. Tuschl , “The G3BP1‐Family‐USP10 Deubiquitinase Complex Rescues Ubiquitinated 40S Subunits of Ribosomes Stalled in Translation From Lysosomal Degradation,” Molecular Cell 77, no. 6 (2020): 1193–1205. e5, 10.1016/j.molcel.2019.12.024. 31981475

[94] F. Buttgereit and M. D. Brand , “A Hierarchy of ATP‐Consuming Processes in Mammalian Cells,” Biochemical Journal 312, no. pt. 1 (1995): 163–167, 10.1042/bj3120163. 7492307 PMC1136240

[95] S. Tahmasebi , A. Khoutorsky , M. B. Mathews , and N. Sonenberg , “Translation Deregulation in Human Disease,” Nature Reviews Molecular Cell Biology 19, no. 12 (2018): 791–807, 10.1038/s41580-018-0034-x. 30038383

[96] T. McGirr , O. Onar , and S. M. Jafarnejad , “Dysregulated Ribosome Quality Control in Human Diseases,” FEBS Journal 292, no. 5 (2025): 936–959, 10.1111/febs.17217. 38949989 PMC11880988

[97] I. M. Adham , M. A. Sallam , G. Steding , et al., “Disruption of the Pelota Gene Causes Early Embryonic Lethality and Defects in Cell Cycle Progression,” Molecular and Cellular Biology 23, no. 4 (2003): 1470–1476, 10.1128/MCB.23.4.1470-1476.2003. 12556505 PMC141158

[98] A. E. O'Connell , M. V. Gerashchenko , M. F. O'Donohue , et al., “Mammalian Hbs1L Deficiency Causes Congenital Anomalies and Developmental Delay Associated With Pelota Depletion and 80S Monosome Accumulation,” PLoS Genetics 15, no. 2 (2019): 1007917, 10.1371/journal.pgen.1007917. PMC6373978 30707697

[99] M. Terrey , S. I. Adamson , J. H. Chuang , and S. L. Ackerman , “Defects in Translation‐Dependent Quality Control Pathways Lead to Convergent Molecular and Neurodevelopmental Pathology,” eLife 10 (2021): 66904, 10.7554/eLife.66904. PMC8075583 33899734

[100] S. Rimal , Y. Li , R. Vartak , et al., “Inefficient Quality Control of Ribosome Stalling During APP Synthesis Generates CAT‐Tailed Species That Precipitate Hallmarks of Alzheimer's Disease,” Acta Neuropathologica Communications 9, no. 1 (2021): 169, 10.1186/s40478-021-01268-6. 34663454 PMC8522249

[101] Z. Wu , Y. Wang , J. Lim , et al., “Ubiquitination of ABCE1 by NOT4 in Response to Mitochondrial Damage Links Co‐Translational Quality Control to PINK1‐Directed Mitophagy,” Cell Metabolism 28, no. 1 (2018): 130–144. e7, 10.1016/j.cmet.2018.05.007. 29861391 PMC5989559

[102] S. Li , Z. Wu , I. Tantray , et al., “Quality‐Control Mechanisms Targeting Translationally Stalled and C‐Terminally Extended Poly(GR) Associated With ALS/FTD,” PNAS 117, no. 40 (2020): 25104–25115, 10.1073/pnas.2005506117. 32958650 PMC7547246

[103] Y. Ren , Y. Li , and D. Tian , “Role of the ABCE1 Gene in human Lung Adenocarcinoma,” Oncology Reports 27, no. 4 (2012): 965–970, 10.3892/or.2012.1646. 22267055 PMC3583587

[104] J. Gao , M. Jung , C. Mayoh , et al., “Suppression of ABCE1‐Mediated mRNA Translation Limits N‐MYC‐Driven Cancer Progression,” Cancer Research 80, no. 17 (2020): 3706–3718, 10.1158/0008-5472.CAN-19-3914. 32651259

[105] N. K. Sinha , A. Ordureau , K. Best , et al., “EDF1 Coordinates Cellular Responses to Ribosome Collisions,” Elife 9 (2020): 58828, 10.7554/eLife.58828. PMC7486125 32744497

[106] A. C. Vind , G. Snieckute , M. Blasius , et al., “ZAKα Recognizes Stalled Ribosomes Through Partially Redundant Sensor Domains,” Molecular Cell 78, no. 4 (2020): 700–713. e7, 10.1016/j.molcel.2020.03.021. 32289254

[107] L. L. Yan and H. S. Zaher , “Ribosome Quality Control Antagonizes the Activation of the Integrated Stress Response on Colliding Ribosomes,” Molecular Cell 81, no. 3 (2021): 614–628. e4, 10.1016/j.molcel.2020.11.033. 33338396 PMC7867595

[108] L. Wan , S. Juszkiewicz , D. Blears , et al., “Translation Stress and Collided Ribosomes Are Co‐Activators of cGAS,” Molecular Cell 81, no. 13 (2021): 2808–2822. e10, 10.1016/j.molcel.2021.05.018. 34111399 PMC8260207

[109] G. Snieckute , A. V. Genzor , A. C. Vind , et al., “Ribosome Stalling Is a Signal for Metabolic Regulation by the Ribotoxic Stress Response,” Cell Metabolism 34, no. 12 (2022): 2036–2046. e8, 10.1016/j.cmet.2022.10.011. 36384144 PMC9763090

[110] A. Garzia , C. Meyer , and T. Tuschl , “The E3 Ubiquitin Ligase RNF10 Modifies 40S Ribosomal Subunits of Ribosomes Compromised in Translation,” Cell Reports 36, no. 5 (2021): 109468, 10.1016/j.celrep.2021.109468. 34348161

[111] J. Geng , T. P. Khaket , J. Pan , et al., “Deregulation of ER‐Mitochondria Contact Formation and Mitochondrial Calcium Homeostasis Mediated by VDAC in Fragile X Syndrome,” Developmental Cell 58, no. 7 (2023): 597–615. e10, 10.1016/j.devcel.2023.03.002. 37040696 PMC10113018

[112] Y. Huang , B. Liu , S. C. Sinha , S. Amin , and L. Gan , “Mechanism and Therapeutic Potential of Targeting cGAS‐STING Signaling in Neurological Disorders,” Molecular Neurodegeneration 18, no. 1 (2023): 79. 37941028 10.1186/s13024-023-00672-x PMC10634099

[113] N. Samson and A. Ablasser , “The cGAS–STING Pathway and Cancer,” Nature Cancer 3, no. 12 (2022): 1452–1463. 36510011 10.1038/s43018-022-00468-w

[114] M. Li , Y. Tang , X. Zuo , S. Meng , and P. Yi , “Loss of Ras GTPase‐Activating Protein SH3 Domain‐Binding Protein 1 (G3BP1) Inhibits the Progression of Ovarian Cancer in Coordination With Ubiquitin‐Specific Protease 10 (USP10),” Bioengineered 13 (2022): 721–734. 34967276 10.1080/21655979.2021.2012624 PMC8805976

[115] B. Qin , X. Chen , F. Wang , and Y. Wang , “DUBs in Alzheimer's Disease: Mechanisms and Therapeutic Implications,” Cell Death Discovery 10, no. 1 (2024): 475. 39562545 10.1038/s41420-024-02237-3 PMC11576995

[116] L. Tao , X. Liu , X. Jiang , et al., “USP10 as a Potential Therapeutic Target in Human Cancers,” Genes 13, no. 5 (2022): 831. 35627217 10.3390/genes13050831 PMC9142050

[117] U. Bhattacharya , F. Neizer‐Ashun , P. Mukherjee , and R. Bhattacharya , “When the Chains Do Not Break: The Role of USP10 in Physiology and Pathology,” Cell Death & Disease 11, no. 12 (2020): 1033. 33277473 10.1038/s41419-020-03246-7 PMC7718870

[118] Z. Lin , H. Yang , C. Tan , et al., “USP10 Antagonizes c‐Myc Transcriptional Activation Through SIRT6 Stabilization to Suppress Tumor Formation,” Cell Reports 5, no. 6 (2013): 1639–1649. 24332849 10.1016/j.celrep.2013.11.029 PMC4007576

[119] H. Sidibé , A. Dubinski , and C. Vande Velde , “The Multi‐Functional RNA‐Binding Protein G3BP1 and Its Potential Implication in Neurodegenerative Disease,” Journal of Neurochemistry 157, no. 4 (2021): 944–962. 33349931 10.1111/jnc.15280 PMC8248322

[120] J. Gal , L. Kuang , K. R. Barnett , et al., “ALS Mutant SOD1 Interacts With G3BP1 and Affects Stress Granule Dynamics,” Acta Neuropathologica 132 (2016): 563–576. 27481264 10.1007/s00401-016-1601-x PMC5023729

[121] Z. Monahan , F. Shewmaker , and U. B. Pandey , “Stress Granules at the Intersection of Autophagy and ALS,” Brain Research 1649 (2016): 189–200. 27181519 10.1016/j.brainres.2016.05.022 PMC5055418

[122] E. Chen and S. Joseph , “Fragile X Mental Retardation Protein: A Paradigm for Translational Control by RNA‐binding Proteins,” Biochimie 114 (2015): 147–154. 25701550 10.1016/j.biochi.2015.02.005 PMC4458461

[123] C. Bagni and R. S. Zukin , “A Synaptic Perspective of Fragile X Syndrome and Autism Spectrum Disorders,” Neuron 101, no. 6 (2019): 1070–1088. 30897358 10.1016/j.neuron.2019.02.041 PMC9628679

[124] I. Lytvynenko , H. Paternoga , A. Thrun , et al., “Alanine Tails Signal Proteolysis in Bacterial Ribosome‐Associated Quality Control,” Cell 178, no. 1 (2019): 76–90. e22, 10.1016/j.cell.2019.05.002. 31155236 PMC6642441

[125] C. S. Sitron and O. Brandman , “CAT Tails Drive Degradation of Stalled Polypeptides on and off the Ribosome,” Nature Structural & Molecular Biology 26, no. 6 (2019): 450–459, 10.1038/s41594-019-0230-1. PMC6554034 31133701

[126] C. S. Sitron , J. H. Park , J. M. Giafaglione , and O. Brandman , “Aggregation of CAT Tails Blocks Their Degradation and Causes Proteotoxicity in S. cerevisiae,” PLoS ONE 15, no. 1 (2020): 0227841, 10.1371/journal.pone.0227841. PMC6964901 31945107

[127] W. D. Chang , M. J. Yoon , K. H. Yeo , and Y. J. Choe , “Threonine‐rich Carboxyl‐terminal Extension Drives Aggregation of Stalled Polypeptides,” Molecular Cell 84, no. 22 (2024): 4334–4349. e7, 10.1016/j.molcel.2024.10.011. 39488212

[128] Y. J. Choe , S. H. Park , T. Hassemer , et al., “Failure of RQC Machinery Causes Protein Aggregation and Proteotoxic Stress,” Nature 531, no. 7593 (2016): 191–195, 10.1038/nature16973. 26934223

[129] R. Yonashiro , E. B. Tahara , M. H. Bengtson , et al., “The Rqc2/Tae2 Subunit of the Ribosome‐Associated Quality Control (RQC) Complex Marks Ribosome‐Stalled Nascent Polypeptide Chains for Aggregation,” Elife 5 (2016): 11794, 10.7554/eLife.11794. PMC4805532 26943317

[130] D. Khan , A. A. Vinayak , C. S. Sitron , and O. Brandman , “Mechanochemical Forces Regulate the Composition and Fate of Stalled Nascent Chains,” Biorxiv: (2024), 10.1101/2024.08.02.606406. PMC12884557 41475347

[131] T. Udagawa , M. Seki , T. Okuyama , et al., “Failure to Degrade CAT‐Tailed Proteins Disrupts Neuronal Morphogenesis and Cell Survival,” Cell Reports 34, no. 1 (2021): 108599, 10.1016/j.celrep.2020.108599. 33406423

[132] J. Chu , N. A. Hong , C. A. Masuda , et al., “A Mouse Forward Genetics Screen Identifies LISTERIN as an E3 Ubiquitin Ligase Involved in Neurodegeneration,” Proceedings of the National Academy of Sciences of the United States of America 106, no. 7 (2009): 2097–2103, 10.1073/pnas.0812819106. 19196968 PMC2650114

[133] P. B. Martin , Y. Kigoshi‐Tansho , R. B. Sher , et al., “NEMF Mutations That Impair Ribosome‐Associated Quality Control Are Associated With Neuromuscular Disease,” Nature Communications 11, no. 1 (2020): 4625, 10.1038/s41467-020-18327-6. PMC7494853 32934225

[134] S. Dango , N. Mosammaparast , M. E. Sowa , et al., “DNA Unwinding by ASCC3 Helicase Is Coupled to ALKBH3‐Dependent DNA Alkylation Repair and Cancer Cell Proliferation,” Molecular Cell 44, no. 3 (2011): 373–384, 10.1016/j.molcel.2011.08.039. 22055184 PMC3258846

[135] X. Zhou , Y. N. Shang , R. Lu , C. W. Fan , and X. M. Mo , “High ANKZF1 Expression Is Associated With Poor Overall Survival and Recurrence‐Free Survival in Colon Cancer,” Future Oncology 15, no. 18 (2019): 2093–2106, 10.2217/fon-2018-0920. 31257922

[136] S. Costantini , F. Capone , A. Polo , P. Bagnara , and A. Budillon , “Valosin‐Containing Protein (VCP)/p97: A Prognostic Biomarker and Therapeutic Target in Cancer,” International Journal of Molecular Sciences 22, no. 18 (2021): 10177, 10.3390/ijms221810177. 34576340 PMC8469696

[137] X. Bi , T. Jones , F. Abbasi , et al., “Drosophila caliban, a Nuclear Export Mediator, Can Function as a Tumor Suppressor in human Lung Cancer Cells,” Oncogene 24, no. 56 (2005): 8229–8239, 10.1038/sj.onc.1208962. 16103875

[138] Q. Yang and R. Gupta , “Zinc Finger Protein 598 Inhibits Cell Survival by Promoting UV‐induced Apoptosis,” Oncotarget 9, no. 5 (2017): 5906–5918, 10.18632/oncotarget.23643. 29464043 PMC5814183

[139] G. Li , Z. Wang , B. Gao , et al., “ANKZF1 Knockdown Inhibits Glioblastoma Progression by Promoting Intramitochondrial Protein Aggregation Through mitoRQC,” Cancer Letters 591 (2024): 216895, 10.1016/j.canlet.2024.216895. 38670305

[140] S. Gopalakrishna , S. F. Pearce , A. M. Dinan , et al., “C6orf203 is an RNA‐Binding Protein Involved in Mitochondrial Protein Synthesis,” Nucleic Acids Research 47, no. 17 (2019): 9386–9399, 10.1093/nar/gkz684. 31396629 PMC6755124

[141] X. Y. Chen , Y. J. Zhu , J. Deng , et al., “[Combined Oxidative Phosphorylation Deficiency Type 7 Caused by C12orf65 Gene Mutations: A Case Report and Literature Review],” Zhongguo Dang Dai Er Ke Za Zhi = Chinese Journal of Contemporary Pediatrics 27, no. 2 (2025): 205–211, 10.7499/j.issn.1008-8830.2409063. 39962784 PMC11838037

[142] H. Antonicka , E. Ostergaard , F. Sasarman , et al., “Mutations in C12orf65 in Patients With Encephalomyopathy and a Mitochondrial Translation Defect,” American Journal of Human Genetics 87, no. 1 (2010): 115–122, 10.1016/j.ajhg.2010.06.004. 20598281 PMC2896764

[143] H. Shimazaki , Y. Takiyama , H. Ishiura , et al., “A Homozygous Mutation of C12orf65 Causes Spastic Paraplegia With Optic Atrophy and Neuropathy (SPG55),” Journal of Medical Genetics 49, no. 12 (2012): 777–784, 10.1136/jmedgenet-2012-101212. 23188110

[144] M. Wesolowska , G. S. Gorman , C. L. Alston , et al., “Adult Onset Leigh Syndrome in the Intensive Care Setting: A Novel Presentation of a C12orf65 Related Mitochondrial Disease,” Journal of Neuromuscular Diseases 2, no. 4 (2015): 409–419, 10.3233/JND-150121. 27858754 PMC5240610

[145] A. C. Nica and E. T. Dermitzakis , “Expression Quantitative Trait Loci: Present and Future,” Philosophical Transactions of the Royal Society of London. Series B, Biological Sciences 368, no 1620 (2013): 20120362, 10.1098/rstb.2012.0362. 23650636 PMC3682727

[146] B. J. Schmiedel , C. Gonzalez‐Colin , V. Fajardo , et al., “Single‐Cell eQTL Analysis of Activated T Cell Subsets Reveals Activation and Cell Type‐Dependent Effects of Disease‐Risk Variants,” Science Immunology 7 (2022): abm2508, 10.1126/sciimmunol.abm2508. PMC9035271 35213211

[147] B. Hu , R. He , K. Pang , et al., “High‐Resolution Spatially Resolved Proteomics of Complex Tissues Based on Microfluidics and Transfer Learning,” Cell 188, no. 3 (2025): 734–748. e22, 10.1016/j.cell.2024.12.023. 39855194

[148] A. Marintchev and G. Wagner , “Translation Initiation: Structures, Mechanisms and Evolution,” Quarterly Reviews of Biophysics 37 (2004): 4, 10.1017/S0033583505004026. 16194295

[149] E. Razumova , A. Makariuk , O. Dontsova , N. Shepelev , and M. Rubtsova , “Structural Features of 5′ Untranslated Region in Translational Control of Eukaryotes,” International Journal of Molecular Sciences 26, no. 5 (2025): 1979. 40076602 10.3390/ijms26051979 PMC11900008

[150] R. J. Jackson , C. U. Hellen , and T. V. Pestova , “The Mechanism of Eukaryotic Translation Initiation and Principles of Its Regulation,” Nature Reviews Molecular Cell Biology 11, no. 2 (2010): 113–127, 10.1038/nrm2838. 20094052 PMC4461372

[151] Y. J. Tseng , A. Krans , I. Malik , et al., “Ribosomal Quality Control Factors Inhibit Repeat‐Associated Non‐AUG Translation From GC‐Rich Repeats,” Nucleic Acids Research 52, no. 10 (2024): 5928–5949, 10.1093/nar/gkae137. 38412259 PMC11162809

[152] J. D. Cleary , A. Pattamatta , and L. P. W. Ranum , “Repeat‐Associated Non‐ATG (RAN) Translation,” Journal of Biological Chemistry 293, no. 42 (2018): 16127–16141, 10.1074/jbc.R118.003237. 30213863 PMC6200949

[153] A. Sriram , J. Bohlen , and A. A. Teleman , “Translation Acrobatics: How Cancer Cells Exploit Alternate Modes of Translational Initiation,” EMBO Reports 19, no. 10 (2018): 45947, 10.15252/embr.201845947. PMC6172470 30224410

